# Safety of low-dose subcutaneous recombinant interleukin-2: systematic review and meta-analysis of randomized controlled trials

**DOI:** 10.1038/s41598-019-43530-x

**Published:** 2019-05-09

**Authors:** Seyed Hamidreza Mahmoudpour, Marius Jankowski, Luca Valerio, Christian Becker, Christine Espinola-Klein, Stavros Konstantinides, Kurt Quitzau, Stefano Barco

**Affiliations:** 1 Center for Thrombosis and Hemostasis (CTH), University Medical Center of the Johannes Gutenberg University, Mainz, Germany; 2 Institute for Medical Biostatistics, Epidemiology, and Informatics (IMBEI), Department of Biometry and Bioinformatics, University Medical Center of the Johannes Gutenberg University, Mainz, Germany; 3 Department of Dermatology, University Medical Center of the Johannes Gutenberg University, Mainz, Germany; 4 Center for Cardiology, Cardiology I, University Medical Center of the Johannes Gutenberg University, Mainz, Germany; 5 Center for Translational Vascular Biology (CTVB), University Medical Center of the Johannes Gutenberg University, Mainz, Germany; 6 Department of Cardiology, Democritus University of Thrace, Alexandroupolis, Greece

**Keywords:** Drug safety, Adverse effects

## Abstract

Standard-dose intravenous recombinant interleukin-2 (rIL-2) is indicated for the treatment of some subtypes of cancer; however, severe adverse events, including venous thromboembolism (VTE), may complicate its administration. Low-dose subcutaneous rIL-2 is being studied for the management of immune-mediated diseases, since it can modulate the immunological response by specifically targeting T regulatory (T_reg_) cells; importantly, it is supposed to cause fewer or no complications. In this systematic review and meta-analysis of phase II-III randomized controlled trials (RCTs), we investigated the safety of low-dose (<6 Million International Unit [MIU]/day) and ultra-low-dose (≤1 MIU/day) rIL-2 for severe adverse events (grade III-V) with a focus on VTE. Data of 1,321 patients from 24 RCTs were analysed: 661 patients were randomized to the rIL-2 arm (on top of standard of care) and 660 patients to standard of care alone or placebo. Two studies reported higher rates of thrombocytopenia in the low-dose rIL-2 arm. Ultra-low-dose rIL-2 was reported to be well tolerated in 6 studies with a negligible rate of severe adverse events. Symptomatic VTE events were not reported in any of the study arms (absolute risk difference 0% [95%CI −0.1%; +0.1%]). Our results may facilitate the study and introduction in clinical practice of low-dose rIL-2 for potentially new indications.

## Introduction

Interleukin 2 (IL-2) is required for the activation, growth, and differentiation of several families of immune cells, including T lymphocytes, B lymphocytes, and natural killer (NK) cells^1,2^. Human recombinant IL-2 (rIL-2), the highly purified protein used in clinical practice, has the same biological activity of the native molecule^3^. Current main indications of rIL-2 include the treatment of metastatic renal cell carcinoma and melanoma^4,5^, for which high-dose intravenous regimens (e.g. above 50 Million International Unit [MIU]/8 hours) are recommended^6^.

In recent years, it has become clear that the immunological effects of rIL-2 are dose dependent. The subcutaneous administration of *low- or ultra-low-dose* rIL-2 (e.g below 5.4 MIU every 8 hours, or a cumulative dose below 52.5 MIU) exerts distinctive immunological effects *in vivo*, which may benefit not only the cancer patients for which *high-dose* rIL2 is already indicated, but also selected groups of patients with other conditions. In fact, low- and ultra-low dose rIL-2 affect the maintenance and proliferation of functional T regulatory cells (T_reg_) with apparently positive effects on the course of immune-mediated diseases, such as type I diabetes^7^, systemic lupus erythematosus^8,9^, immune thrombocytopenia^10^, vasculitis induced by the hepatitis C virus (HCV)^11^, and other autoimmune diseases^12^. Moreover, regulatory T cells, which are reliant on IL-2 levels, might positively influence the process of thrombus resolution after acute venous thromboembolism (VTE)^13,14^.

Serious safety concerns have been raised around the use of high-dose IL-2, which has been reported to potentially cause acute thromboembolic events, including VTE^15,16^, as well as cardiac, cerebral, and hepatic venous thrombosis^17–19^, as also reported in the product monograph^20^. In the Evaluation of Subcutaneous Proleukin in a Randomized International Trial (ESPRIT) study, the hazard ratio for arterial and venous events with IL-2 administered on top of antiretroviral therapy (vs. antiretroviral therapy alone) was 2.80 (95% Confidence Interval [95% CI] 1.53–5.15) with four-month rates of 8.6% and 3.7%, respectively^16^. The most frequent type of event was deep vein thrombosis, occurring in 10 (2.1%) and 2 (0.5%) patients, respectively^16^. In a pathophysiological perspective, such increased risk in thrombotic events is explained by the fact that IL-2 can increase platelet adherence^21^ and activate the intrinsic coagulation pathway^22^. Furthermore, animal models suggest that rIL-2 inhibits the expression and activity cytochromes and transporters involved in the absorption and metabolism of oral anticoagulants^23,24^. Other severe adverse events (AE) reported in patients administered high-dose rIL-2 include capillary leak syndrome, sepsis, and autoimmune reactions^20^.

In contrast, preliminary findings suggest that low-dose rIL-2 may be better tolerated and characterized by lower AE rates compared with higher doses^25^. However, in the absence of any comprehensive assessment of its safety, the administration of rIL-2 for research purposes to patients diagnosed with conditions characterized by an intrinsic high risk of severe adverse events and venous thromboembolism should be cautious^26^. In our systematic review and meta-analysis, we investigated the safety of low- or ultra-low-dose subcutaneous rIL-2 administration in humans.

## Results

In our systematic review, we searched the literature and meta-analysed the results of phase II-III randomized controlled trials in which patients were assigned to receive either rIL-2 on top of the standard of care or standard of care alone.

### Study selection

Following the predefined search strategy and after removal of duplicated records, our literature search identified 1,672 records. A total of 24 articles were selected, after the full-text evaluation^27–50^. Supplementary Fig. S1 summarizes the process of study selection and the reasons for study exclusion. The search for unpublished randomized clinical trials on the topic resulted in the evaluation of 79 additional studies that were registered on the database (http://www.clinicaltrials.gov): however, none of them met the inclusion criteria, since they were single-arm studies (n = 45), non-randomized (n = 10), used a high dose of rIL-2 (n = 11), studied another exposure (n = 4), or already published and included in our study (n = 9).

### Characteristics of the included studies and quality assessment

The sample size of the 24 included studies ranged from 10 to 241 patients for a total number of 1,321 patients enrolled^27–50^: of these, 661 were randomized to receive low-dose rIL-2 (on top of the standard of care) and 660 the standard of care alone or the placebo. One trial included patients with autoimmune disease (Type 1 diabetes)^33^, eight trials focused on patients with active cancer (usually at stage II or higher)^36,38,40–44,49^, and 15 trials enrolled patients with infectious diseases, more often by human immunodeficiency virus (HIV) (n = 11)^27,28,31,32,35,37,39,45,47,48,50^. The average follow-up time ranged from 7 to 973 days.

Although the therapeutic dose of rIL-2 for the approved indications (renal carcinoma and melanoma) is calculated based on weight (kg) or body surface (m^2^), 19 of the 24 included trials in our review used a fixed low dose of rIL-2, ranging from 0.33 MIU/day to 6 MIU/day. The highest daily rIL-2 dose (6 MIU/day) was administered in three trials enrolling HIV positive patients^28,32,50^ and in one trial on melanoma patients^41^. Four trials administered various dosages of rIL-2^28–31^. The detailed general characteristics of the included studies are summarised in Table 1.

**Table 1:** General characteristics of the included studies.

Study	N	Age (years)	Sex, % male	Disease (C/I/A)	Intervention arm	Comparator arm	Follow-up (days)	IL-2 dose
Amendola^27^	22	µ = 36	55	HIV (I)	rIL-2 + ART	ATR	196	1 MIU/day
Arnó^28^	25	µ = 35 (23–48)	80	HIV (I)	rIL-2 + ART	ART	252	6 MIU/day first cycle, reduced to 3 MIU/day
Artillo^29^	30	µ = 29	NA	Chronic HBV hepatitis (I)	rIL-2	Placebo	140	0.9–3.6 MIU/day
Bruch^30^	37	µ = 45 (23–60)	83	Chronic HBV hepatitis (I)	rIL-2 + IFN alpha-2b	IFN alpha-2b	243	0.3–1.5 MCU/day
Carr^31^	115	µ = 38	98	HIV (I)	PEG rIL-2 + ART	ART	~ 280 (median)	0.5–4.25 MIU/day
De Paoli^32^	22	µ = 40	77	HIV (I)	rIL-2 + RTIs + Indinavir	RTIs + Indinavir	672	6 MIU/day
Hartemann^33^	25	µ = 31 (19–51)	NA	Type 1 diabetes (A)	rIL-2	Placebo	60	0.33–3 MIU/day
Johnson^34^	110	µ = 27	68	Confirmed tuberculosis (HIV-) (I)	rIL-2 + standard chemotherapy	Standard chemotherapy	365	450,000 IU/day
Lalezari^35^	115	µ = 41	96	HIV (I)	rIL-2 + ART	ART	183	1.2 MIU/m^2^/day
Li^36^	40	µ = 47 (20–65)	NA	Breast cancer (C)	rIL-2 + postoperative standard care	postoperative standard care	7	1 MIU/day
Losso^37^	71	µ = 34	79	HIV (I)	rIL-2 + ART	ART	168	3 MIU/day
Mantovani^38^	33	µ = 56 (38–72)	87	Head and neck squamous-cell carcinoma (C)	rIL-2 + classical Al Sarraf treatment	Classical Al Sarraf treatment	~487	4.5 MIU/day
Marchetti^39^	22	µ = 36 (28–55)	72	HIV (I)	rIL-2 + HAART	HAART	336	3 MIU/day
Nichols^40^	25	µ = 72 (50–88)	64	Colorectal cancer, Dukes’ stage A-D (C)	rIL-2 + Standard surgery	Standard surgery	10	1.8 MIU/m^2^/ twice daily
Nicholson^41^	41	µ = 47	NA	Melanoma (C)	rIL-2 + SRL172	SRL172	183	6 MIU/day
Perillo^42^	32	µ = 48 (33–63)	0	Breast cancer/Ovarian cancer(C)	rIL-2 + G-CSF + EPO	G-CSF + EPO	100	200,000 IU/m^2^/day
Procopio^43^	128	M = 63 (52–69)	74	Renal cell carcinoma (C)	rIL-2 + oral sorafenib	Oral sorafenib	821	4.5 MIU/day
Ridolfi^44^	241	M = 62 (32–76)	76	Non-small-cell lung cancer (C)	rIL-2 + gemcitabine + cisplatin	Gemcitabine + cisplatin	973	3 MIU/day
Ruxrungtham^45^	72	µ = 31 (19–58)	32	HIV (I)	rIL-2 + ART	ART	168	3 MIU/day
Shen^46^	50	µ = 45	62	Multidrug-resistant tuberculosis, resistant to isoniazid and rifampicin (I)	rIL-2 + multidrug chemotherapy	Multidrug chemotherapy	730	500,000 IU/day
Smith^47^	44	µ = 41 (22–63)	93	HIV (I)	rIL-2 + placebo vaccine	placebo vaccine	25	1.2 MIU/m^2^/day
					rIL-2 + vCP1452 vaccine	vCP1452 vaccine		
Vogler^48^	115	M = 38 (24–70)	85	HIV (I)	rIL-2 + ART	ART	168	1 MIU/day
Woodson^49^	40	µ = 55 (25–77)	62	Melanoma (C)	rIL-2 + six peptide vaccinations	Six peptide vaccinations	28	3 MIU/m^2^/day
Zanussi^50^	10	µ = 30 (23–54)	70	HIV (I)	rIL-2 + HAART	HAART	168	6 MIU/day

µ: Mean (range); M: median (range); (C/I/A): Cancer/Infection/autoimmune disease; MIU: Million international units, IU: International units; MCU: Million Cetus units (Cetus was the company which was first involved in the development of rIL-2); NA: Not available; HAART: Highly active antiretroviral therapy; RTI: Reverse-transcriptase inhibitors; PEG rIL-2: Polyethylene glycol-modified interleukin-2; ART: Anti-retroviral therapy; G-CSF: Granulocyte-colony stimulating factor; EPO: Erythropoietin; IFN: Interferon; HBsAG: Surface antigen of the hepatitis B virus; HBV: Hepatitis B virus; rIL-2: recombinant interleukin 2; Classical Al Sarraf treatment: (100 mg/m2 cisplatin i.v. as a 60-min infusion on day 1, with a standard pre- and post-hydration protocol with forced diuresis by 250 ml 18% mannitol, plus 1000 mg m^−2^ day^−1^ 5-fluorouracil on days 1–5 (120 h) as a continuous infusion).

The assessment of quality and risk of bias showed that three studies were classified to a low risk of bias^33,34,43^. Risk of selection bias was not detected in only one of the included studies, due to the manner in which participant were screened for eligibility and enrolled^47^. Since most of the included studies were open label trials, the lack of blinding of participants and personnel carried a high or unclear risk of bias; however, blinding of outcome was properly conducted in more than 50% of studies. Risk of incomplete outcome was assessed as low in around 50% of the included studies while we evaluated the risk of selective reporting bias as high in 50% of included studies (Table 2 and Supplementary Fig. S2).

**Table 2:** Summary of the risk of bias for the assessment of severe adverse effects.

Study	Method of assessment	Description	Exclusion of patients from the AE analysis	Presence of numerical data by intervention group
Amendola^27^	1 (Not specified)	No description of safety assessment provided; the only blood sampling was performed at baseline and at the end of the study.	Not applicable	No
Arnó^28^	3 (Prospective monitoring)	Patients were monitored throughout the study for safety; safety parameters included hematologic, renal, and hepatic routine tests. Based on a standardized and predefined grading scale	No	Yes
Artillo^29^	3 (Prospective monitoring)	The patients were studied every 4 weeks during treatment and 12 weeks post-treatment with a clinical examination and blood samples at each visit.	No	No
Bruch^30^	2 (Retrospective or possibly prospective monitoring)	Blood samples were taken at monthly intervals during treatment. Side effects are generically described.	No	No
Carr^31^	3 (Prospective monitoring)	Adverse events and laboratory tests were assessed during each treatment cycle, and two interim analyses to evaluate safety were specified in the study protocol, based on a standardized and predefined grading scale.	No	Yes
De Paoli^32^	2 (Retrospective or possibly prospective)	No predefined study visits is described, but grade toxicity of at least grade 2 guided dose reductions, suggesting some degree of prospective monitoring. Based on a standardized and predefined grading scale	No	Yes
Hartemann^33^	3 (Prospective monitoring)	Safety was assessed at predefined visits by a history taking and physical examination procedure described in detail, based on a standardized and predefined grading scale.	No	Yes
Johnson^34^	3 (Prospective monitoring)	Adverse events assessed at all study visits (thrice weekly) and injection sites twice daily; laboratory tests were assessed weekly during the study treatment.	No	Yes
Lalezari^35^	3 (Prospective monitoring)	Adverse events assessed at all study visits (10 out of 10), laboratory tests on predefined subset of study visits (5 out of 10); based on a standardized and predefined grading scale.	No	Yes
Li^36^	2 (Retrospective or possibly prospective monitoring)	Treatment with rIL-2 was performed post-modified radical mastectomy for primary breast cancer for 5 days and peripheral blood samples were collected on days 0, +1, +3, and +7. It is not explicitly stated whether patients were hospitalized during the whole treatment duration.	No	Yes
Losso^37^	2 (Retrospective or possibly prospective monitoring)	Control patient visits were performed and toxicity guided dose adjustments. However, no predefined study visits dates or intervals are specified; recipients of rIL-2 therapy underwent more frequent safety monitoring than control patients; and that grade IV toxicities and serious adverse events were recorded, while an overview of all toxicities was made retrospectively upon study completion. This suggests that monitoring was only in part prospective. Reporting was based on a standardized and predefined grading scale.	No	Yes
Mantovani^38^	2 (Retrospective or possibly prospective monitoring)	Patients received a full clinical and laboratory multi-specialist evaluation only at the beginning and the end of the study period, but monitoring of toxicity during chemotherapy cycles is mentioned. Based on a standardized and predefined grading scale.	No	Yes
Marchetti^39^	3 (Prospective monitoring)	Clinical evaluation including rIL-2 side effects and laboratory tests were assessed at predefined study visits; based on a standardized and predefined grading scale.	No	Yes
Nichols^40^	2 (Retrospective or possibly prospective monitoring)	Toxicity monitoring is not mentioned in the Methods. However, the treatment was perioperative, suggesting in-hospital monitoring, and a dedicated paragraph on toxicity was present in the Results.	No	Yes
Nicholson^41^	2 (Retrospective or possibly prospective monitoring)	Blood samples were drawn before and at the end of treatment; a number of patients received additional five treatment doses and were re-evaluated clinically and by imaging after the fifth dose. However, ‘flu-like’ symptoms on treatment days are mentioned, suggesting at least partial continuous monitoring.	No	No
Perillo^42^	2 (Retrospective or possibly prospective monitoring).	No description of safety assessment is provided, but patients were hospitalized during the whole study (both treatments lasted from day +1 to +12, while the range of hospital stay in days was 16–21 in the control arm, 15–23 in the rIL-2 arm).	No	Yes
Procopio^43^	2 (Retrospective or possibly prospective monitoring)	Based on a standardized and predefined grading scale.	No	Yes
Ridolfi^44^	3 (Prospective monitoring)	Clinical toxicity assessment was performed at baseline, before each chemotherapy course and every 3 months during follow-up based on a standardized and predefined grading scale.	No	Yes
Ruxrungtham^45^	2 (Retrospective or possibly prospective monitoring)	No predefined study visits are mentioned; however, it is specified that grade IV toxicities and serious adverse events were recorded. Based on a standardized and predefined grading scale.	No	No
Shen^46^	3 (Prospective monitoring)	Adverse events counted during treatment; and laboratory tests assessed monthly during treatment.	No	Yes
Smith^47^	2 (Retrospective or possibly prospective monitoring)	While no explicit mention is made of safety or adverse effects monitoring, in at least part of the study period visits were timed independently of treatment administration, suggesting that these visits were meant for safety evaluation rather than just treatment administration. Based on a standardized and predefined grading scale.	No	Yes
Vogler^48^	3 (Prospective monitoring)	Patients were trained to inject themselves, but they could report any toxicity they found intolerable to allow for dose reduction. The protocol specified an interim analysis of toxicity. Based on a standardized and predefined grading scale.	No	Yes
Woodson^49^	3 (Prospective monitoring)	Both adverse events and laboratory tests assessed weekly, the former also based on a daily symptom diary kept by patients. Based on a standardized grading scale.	No	Yes
Zanussi^50^	2 (Retrospective or possibly prospective monitoring)	No pre-defined study visits are mentioned other than tumor response assessment with maximal frequency of 8 weeks. However, dose adaptation and a protocol amendment based on toxicity are described in detail. Based on a standardized grading scale.	No	No

Assessment of adverse events is classified in three categories: (1) Not specified: no mention of predefined study visits or intervals other than total duration of follow-up; no mention of a procedure for monitoring safety or adverse effects is made other than the possible use of a standardized grading; (2) Retrospective or possibly prospective monitoring: predefined study visits or their intervals are specified for laboratory and clinical assessment, although no explicit mention is made of regular monitoring of adverse effects or safety; patient checklist or diaries may be mentioned as a specific tool. (3) Prospective monitoring: it is explicitly stated that adverse events or safety were assessed during predefined study visits or interim analyses of which the timing is stated; physical examination may be mentioned as a specific tool. Adapted from Loke *et al*.^61^. AE: adverse event; rIL-2: recombinant interleukin 2.

### Safety of rIL-2

Of the 24 included studies, 14 (58%) reported no grade III-V AEs in either the intervention or the control arm^27–30,33,34,36,39–42,45,46,49^. In eight studies, no statistically significant difference was observed between the intervention and comparator arms^32,35,37,38,43,47,48,50^. None of the included trials reported any major bleeding complications. Two studies reported higher rates of thrombocytopenia among rIL-2-treated patients. In one of these studies, that was conducted in HIV patients, thrombocytopenia was reported only in the rIL-2 arm^31^; in the other study, that was conducted in lung cancer patients, it was reported in both arms, with a statistically significantly higher rate in the rIL-2 arm^44^. The summary of grade III-V AEs is presented in Table 3.

**Table 3:** Reported severe adverse events (grade III-IV-V).

Study	Grade III	Grade IV-V	Type of AE	Notes
***Infectious diseases***				
Amendola^27^	NA	NA	Not reported	NA
Arnó^28^	=	=	None reported	NA
Artillo^29^	=	=	None reported	1 patient in rIL-2 group went out of the study due to the allergic reaction
Bruch^30^	=	=	None reported	NA
Carr^31^	+	=	Thrombocytopenia	Grade IV: 4% (rIL-2)
De Paoli^32^	=	=	Hyperbilirubinemia, neurological toxicity	Grade III: 27% (rIL-2) vs 20%. Grade IV–V: none.
Johnson^34^	=	=	None reported	NA
Lalezari^35^	=	=	Myocardial infarction, optic neuritis, pneumonia	Grade III: 8% (rIL-2) vs 6%. Grade IV: none. Grade V: death (n=1, sudden unexplained death; comparator arm)
Losso^37^	=	=	Erythema multiforme	Grade IV AE (n=1; rIL-2 arm). Grade V: (n=1 possibly due to sepsis; comparator arm)
Marchetti^39^	=	=	None reported	NA
Ruxrungtham^45^	=	=	None reported	The frequency and severity of toxicities was dose-dependent. No grade III or higher AE were observed in low dose rIL-2
Shen^46^	=	=	None reported	NA
Smith^47^	=	=	Allergic reaction, neutropenia	Grade III: 7.1% (rIL-2) vs 2.5%. Grade IV–V: none.
Vogler^48^	=	=	Hypertriglyceridemia, agitation	Grade III: 24% (rIL-2) vs 15%. Grade IV–V: 3% (rIL-2).
Zanussi^50^	=	=	Hyperbilirubinemia	Grade III: 2 episode (rIL-2) vs 0 episode. Grade IV–V: none
***Autoimmune diseases***				
Hartemann^33^	=	=	None reported	NA
*Cancer*				
Li^36^	=	=	None reported	NA
Mantovani^38^	=	=	Cardiac toxicity, erythema, fever	Death due to haematological toxicity (n=1; comparator arm). Death due to cardiac toxicity (n=1; rIL-2 arm)
Nichols^40^	=	=	None reported	NA
Nicholson^41^	=	=	None reported	NA
Perillo^42^	=	=	None reported	NA
Procopio^43^	=	=	skin, gastrointestinal	Grade III or higher: 38% (rIL-2) vs 25%.
Ridolfi^44^	=	+	Thrombocytopenia, nausea, diarrhea, myelosuppression	Higher rate of thrombocytopenia in the rIL-2 group: 25.5% vs 9.9%
Woodson^49^	=	=	None reported	NA

Symbol ‘=’: the authors did not report significant difference between groups; ‘+’: significant difference (rIL-2 worse), as reported in the individual study; ‘-’: significant difference (rIL-2 better), as reported in the individual study.

AE: Adverse event; NA: not applicable; rIL-2: recombinant interleukin 2

*Grade refers to the severity of the AE. The terminology criteria for adverse events (CTCAE) displays Grades 1 through 5 with unique clinical descriptions of severity for each AE based on this general guideline. Grade I: Mild; asymptomatic or mild symptoms; clinical or diagnostic observations only; intervention not indicated. Grade II: Moderate; minimal, local or non-invasive intervention indicated; limiting age-appropriate instrumental Activities of Daily Living (ADL). Grade III: Severe or medically significant but not immediately life-threatening; hospitalization or prolongation of hospitalization indicated; disabling; limiting self-care ADL. Grade IV: Life-threatening consequences; urgent intervention indicated. Grade V: Death related to AE.

A total of six studies investigated the effects of an ultra-low-dose of rIL-2 (≤1 MIU/day)^27,33,34,36,46,48^. Of these, two did not report any AE^27,36^, which were reported in long-term follow-up studies to occur in similar proportions in rIL-2 and placebo or comparator. Local reactions at the injection sites were the most frequently reported grade I-II AE in two studies, with a prevalence of 50% to 100% among patients with type I diabetes and tuberculosis, respectively^33,34^. Other reactions included influenza-like syndrome, headache, and dyspnoea (Supplementary Table S1).

None of the included interventional studies reported any thromboembolic complications. Therefore, the pooled absolute risk difference between the rIL-2 arm and controls essentially was 0 (95% CI: −0.1%; +0.1%) (Supplementary Fig. S3). In order to minimize the risk of underreporting, we contacted all 17 corresponding authors of the included research articles for whom the e-mail address was available^27,28,31–34,36–39,41,43,44,46–49^: seven of them were able to retrospectively review the study case report forms and data, and confirmed that no thromboembolic events were observed (total number of 626 patients from those studies)^34,39,41,43,44,47,49^. Ten authors did not answer to our e-mails or their e-mail address was no longer active.

## Discussion

Our systematic review and meta-analysis of the results of phase II-III clinical trials showed that the use of subcutaneous low-dose rIL-2 on top of the standard of care is well tolerated and does not appear to increase the risk of developing thromboembolic events in patients with various conditions, notably HIV, tuberculosis, autoimmune diseases, or cancer. The safety profile of rIL-2 appeared particularly favourable when rIL-2 was given at ultra-low dose. Two trials reported a possible association of rIL-2 with thrombocytopenia after multiple cycles of treatment in populations at an intrinsic higher risk for this complication, such as cancer or HIV patients.

Since rIL-2 can selectively expand T-lymphocytes populations, it had been tested experimentally in a number of chronic diseases, including chronic infections or cancers, in which T-cell activation plays a pathophysiological and possibly therapeutic role. The initial lack of positive findings prompted researchers to progressively increase doses and, in this perspective, a number of interventional studies were conducted more than two decades ago. However, this approach led to higher rate and severity of AE with no significant gains in terms of efficacy^51^. Recently, it has been observed that this might have been a misleading approach: high doses of rIL-2 are more than sufficient to saturate both the high- and the intermediate-affinity rIL-2 receptor, but they also lead to massive release of proinflammatory cytokines and directly trigger the capillary-leak syndrome^52^. In contrast, the ultra-low doses of rIL-2 seem sufficient to trigger T_reg_ lymphocytes expansion, probably because of its affinity for the rIL-2 heterotrimeric receptor components, with higher doses providing no added value^53,54^. This cell expansion might be clinically relevant: a recent human study suggested that administration of low-dose rIL-2 to patients with active systemic lupus erythematosus changes the proportions of T effector memory cells (T_effector_) and T_reg_, which in turn reduces disease activity^55^; and animal studies have shown that a selective expansion and activation of T_reg_ lymphocytes as achieved by ultra-low rIL-2 may influence thrombus resolution^13^. In this perspective, our finding that ultra-low-dose rIL-2 is safe appears promising with a view to future studies in patients with or at risk for VTE.

Although we aggregated all the available data on the safety of low-dose rIL-2, the limited number of individual patients in our analyses should be acknowledged, which may hinder the clinical interpretation of the results. This is inevitable because of the novelty of the low-dose concept of rIL-2 and because the proposed indications include relatively rare diseases. In our systematic review, selective reporting was the most prominent source of bias. We focused on AEs rather than on efficacy outcomes of the trials; venous thrombotic events, in particular, have been shown to be often underreported in randomized trials^56^. Accordingly, we attempted to minimize this risk by directly contacting all the authors of the studies included in our systematic review and asking for confirmation that no event of interest had occurred. It must be noted that we did not meta-analyse the overall rates of AEs, as studies varied substantially in the nature and severity of the diseases studied as well as in the assessment of AEs, thus preventing any meaningful interpretation of pooled results. The extracted data on all AEs showed that thrombocytopenia was the only severe AE (grade III or higher) reported with significantly higher rates in low-dose rIL-2 than controls in HIV patients: 4% vs 0%, and in lung cancer patients: 25.5% vs. 9.9%^31,44^. Both studies were conducted on patient populations with a very high baseline risk for thrombocytopenia due to the primary diseases, especially in the case of HIV^57^. This specific AE was not observed in studies using ultra-low-dose rIL-2 (Supplementary Table S1)^27,33,34,36,46,48^. Until further evidence in this regard is available, regular monitoring of blood counts should be considered when rIL-2 administration is planned in patients at a higher risk for thrombocytopenia.

In conclusion, the use of low- or ultra-low-dose subcutaneous rIL-2 did not appear to be associated with an increased risk of venous thromboembolic events in randomized controlled trials. Administration of low-dose rIL-2 can be considered safe for clinical and experimental use in humans since an overall low rate of severe adverse events was observed, especially if given at ultra-low dose. It is, however, reasonable to monitor platelet count in patients at risk for thrombocytopenia, e.g. in the presence of active cancer or HIV, in particular if multiple treatment cycles with rIL-2 are envisaged.

## Methods

### Study selection

The systematic search was conducted in MEDLINE (via PubMed), Google Scholar, the database of ‘http://www.clinicaltrials.gov’, and in the Cochrane Collaboration database, from inception to April 7^th^, 2018, without any language restrictions. The full search strategy is available as Supplementary Material. We complemented this search by manually reviewing the references of retrieved articles, relevant review papers, guidelines documents, and the grey literature. Authors of the selected studies were contacted by electronic mail if there was ambiguity about original data and to receive confirmation regarding the methods of AE assessment, as well as the characteristics of reported AEs. After removal of duplicates, titles and abstracts of the articles were screened independently by two reviewers (SHM and MJ) for eligibility; disagreements were solved by a third reviewer (SB). The present review was conducted in accordance with the Preferred Reporting Items for Systematic Reviews and Meta-Analyses (PRISMA) statement methodology^58^.

### Search strategy

We included peer-reviewed studies meeting the following criteria:

- Study population composed of adults enrolled into a randomized controlled phase II-III trial investigating the effects of rIL-2 on surrogate laboratory parameters, on clinical outcomes, or both, and administered for any of the following indications: (i) viral or bacterial infection, (ii) solid cancer, or (iii) active autoimmune disease.
- Intervention: subcutaneous rIL-2 given at a low dose on top of the standard of care. Low-dose rIL-2 was defined by a daily total dose not exceeding 6 MIU and/or a cumulative dose not exceeding 60 MIU according to the definition of low-dose rIL-2 provided by Klatzmann and Abbas^25^.
- Comparator: standard of care alone (or placebo).
- Primary outcomes: AE (grading according to the Common Terminology Criteria for Adverse Events) which include acute VTE, defined as objectively diagnosed symptomatic deep vein thrombosis, pulmonary embolism or other major thromboses (cerebral vein thrombosis, splanchnic thrombosis, central catheter thrombosis) as well as major bleeding^59^;
- Length of follow-up: as specified in the original article. If multiple cycles were administered, the primary focus was put on the first rIL-2 treatment cycle.
- Study design: phase II or phase III randomized controlled trials.

### Data extraction

Full-texts of all included studies were retrieved through the library of the Johannes Gutenberg University Mainz or by contacting the authors. The following information was extracted from the included studies: year of publication, patients’ baseline characteristics, sample size, duration of follow-up, disease for which patients were treated, procedure for recording of the AEs, interventions including the administered dose, rate of study outcomes. We extracted data regarding severe AEs (e.g. grade III or higher) which include major bleeding events. We furthermore focused on thromboembolic events. We predefined a subgroup analysis focusing on studies adopting an ultra-low-dose rIL-2 (≤1 MIU/day).

### Quality assessment

Two reviewers (MJ and SB) independently assessed the quality and the risk of bias in accordance to the criteria recommended by the Cochrane Handbook for Systematic Reviews of Interventions Version 5.1.0^60^. Additional details regarding this process are provided as Supplementary Material. Furthermore, we assessed specifically the risk of bias for evaluation and reporting of AE as recommended by the Cochrane Adverse Effects Methods Group^60,61^. The presence of the following items was evaluated: description of the method used for the assessment of AEs, exclusion of patients from the AE analysis, and presence of numerical data by intervention group.

### Statistical analysis

The number of subjects who developed the outcome of interest was extracted; if no case was recorded, the authors were contacted for confirmation. We relied on data from the original articles when the authors did not reply. Risk differences and 95% CI for developing the VTE were calculated for all studies separately and subsequently pooled using the Mantel-Haenszel random effects model. Heterogeneity of results among studies was tested with the I^2^ measure, which describes the percentage of total variation across studies that is due to heterogeneity rather than chance (I^2^ values > 50% indicate a substantial level of heterogeneity). Review Manager was used to pool the data (RevMan; version 5.3 for Windows; Copenhagen: The Nordic Cochrane Centre, The Cochrane Collaboration, 2014).

## Supplementary information

Supplementary Information

## Data Availability

All data generated or analysed during this study are included in this published article (and its Supplementary Information Files).
