# Supplementary material for: Safety of low-dose subcutaneous recombinant interleukin-2: systematic review and meta-analysis of randomized controlled trials
Source: Sci Rep. 2019 May 9;9:7145. doi: 10.1038/s41598-019-43530-x (PMC6509335; doi:10.1038/s41598-019-43530-x)
Supplement: Supplementary file 1 — Supplementary Information [file 41598_2019_43530_MOESM1_ESM.docx]

*Supplementary Information*

**Safety of low-dose subcutaneous recombinant interleukin-2: systematic review and meta-analysis of randomized controlled trials**

Seyed Hamidreza Mahmoudpour,^1,2^ Marius Jankowski,^1^ Luca Valerio,^1^ Christian Becker,^1,3^ Christine Espinola-Klein,^4,5^ Stavros Konstantinides,^1,6^ Kurt Quitzau,^1^ Stefano Barco^1*^

**Affiliations**

1. Center for Thrombosis and Hemostasis (CTH), University Medical Center of the Johannes Gutenberg University, Mainz, Germany

2. Institute for Medical Biostatistics, Epidemiology, and Informatics (IMBEI), Department of Biometry and Bioinformatics, University Medical Center of the Johannes Gutenberg University, Mainz, Germany

3. Department of Dermatology, University Medical Center of the Johannes Gutenberg University, Mainz, Germany

4. Center for Cardiology, Cardiology I, University Medical Center of the Johannes Gutenberg University, Mainz, Germany

5. Center for Translational Vascular Biology (CTVB), University Medical Center of the Johannes Gutenberg University, Mainz, Germany

6. Department of Cardiology, Democritus University of Thrace, Alexandroupolis, Greece

***Corresponding author**

Stefano Barco, MD, PhD

Center for Thrombosis and Hemostasis (CTH), University Medical Center of the Johannes Gutenberg University; Langenbeckstraße 1 - 401-131, 55131 Mainz, Germany

Tel: +49 6131 17 8251. Fax: +49 6131 17 8461. E-mail: [s.barco@uni-mainz.de](mailto:s.barco@uni-mainz.de)

**Supplementary Table S1. Adverse events (grade I-IV) from clinical trials using ultra-low-dose recombinant IL-2 (less than 1 MIU/day).**

| **Study**  **year** | **Population**  **follow-up** | **rIL-2 dose** | **Type of AE*** | **rIL-2 (ultra-low-dose) arm,**  **% of total (N)** | **Control/placebo arm, % of total (N)** |
| --- | --- | --- | --- | --- | --- |
| Hartemann  2013 ^1^ | Type-1 diabetes    6 days | 330,000 IU/day | Grade I-II (Days 1-6)  Injection-site reaction  Influenza-like syndrome  Diarrhoea  Nausea  Rhinitis  Asthenia, fatigue  Headache, migraine  Throat constriction  Dyspnoea, chest tightness  Grade I-II (Days 7-60)  Asthenia, fatigue  Headache, migraine  Abdominal pain  Grade III–IV  No serious adverse events | N = 6  50%  17%  0%  0%  0%  17%  50%  0%  17%  0%  0%  0%  0% | N = 6  0%  0%  17%  0%  0%  50%  33%  17%  0%  0%  33%  17%  0% |
| Johnson  2003 ^2^ | Tuberculosis  365 days | 450,000 IU/day | Grade I-II  *Local adverse events:*  Pain at injection site  Tenderness on palpation  Erythema  Ecchymosis  Hyperpigmentation  Axillary pain  *Systemic adverse events:*  General  Dermatologic  Endocrine  Gastrointestinal  Genitourinary  Lymphatic  Musculoskeletal  Neurological  Parasitic, infectious  Respiratory  Grade III-IV  *Systemic adverse events:*  General  Genitourinary  Musculoskeletal  Parasitic, infectious  Respiratory | N = 55  49%  87%  96%  18%  100%  2%  9%  60%  2%  22%  13%  24%  58%  2%  20%  75%  69%  2%  2%  2%  2% | N = 55  2%  4%  2%  2%  2%  0%  16%  49%  2%  36%  7%  4%  73%  2%  18%  78%  58%  0%  0%  0%  2% |
| Li  2015 ^3^ | Breast cancer  7 days | 1 MIU/day | Grade I-II  Drug inhalation  Fever  Hypotension | N = 20  0%  20%  5% | N = 20  5%  0%  0% |
| Shen  2013 ^4^ | Tuberculosis  730 days | 500,000 IU/day | Grade I-II  Blood system influence  Hepatic injury  Kidney injury  Gastrointestinal reaction  Muscle and joint pain  Electrolyte abnormalities  Thyroid dysfunction  Grade III–IV  No serious adverse events reported | N = 25  12%  8%  12%  8%  8%  12%  0% | N = 25  0%  16%  12%  16%  16%  8%  4% |
| Vogler  2004 ^5^ | HIV  168 days | 1 MIU/day | Grade II  Laboratory abnormalities:  Hematologic  Hepatic  Creatine phosphokinase  Metabolic  Gastrointestinal  Neuropsychologic  Hearing abnormality  Pulmonary  Skin (mucocutaneous)  General body  Grade III:  Laboratory abnormalities:  Hematologic  Hepatic  Metabolic  Gastrointestinal  Neuropsychologic  Pulmonary  Skin (mucocutaneous)  General body | N = 57  11%  14%  2%  11%  11%  12%  2%  5%  14%  42%  4%  4%  2%  5%  4%  2%  2%  7% | N = 58  5%  4%  0%  14%  9%  16%  0%  7%  9%  21%  2%  7%  5%  2%  0%  2%  2%  7% |

*Adverse events: Grade refers to the severity of the AE. The common terminology criteria for adverse events (CTCAE) displays Grades 1 through 5 with unique clinical descriptions of severity for each AE based on this general guideline: Grade I: Mild; asymptomatic or mild symptoms; clinical or diagnostic observations only; intervention not indicated. Grade II: Moderate; minimal, local or non-invasive intervention indicated; limiting age-appropriate instrumental Activities of Daily Living (ADL). Grade III: Severe or medically significant but not immediately life-threatening; hospitalization or prolongation of hospitalization indicated; disabling; limiting self-care ADL. Grade IV: Life-threatening consequences; urgent intervention indicated. Grade V: Death related to AE. rIL-2: recombinant interleukin 2; AE: adverse event; MIU: million international unit; IU: international unit; HIV: human immunodeficiency virus;

**Supplementary Figure S1. Study selection.**

**
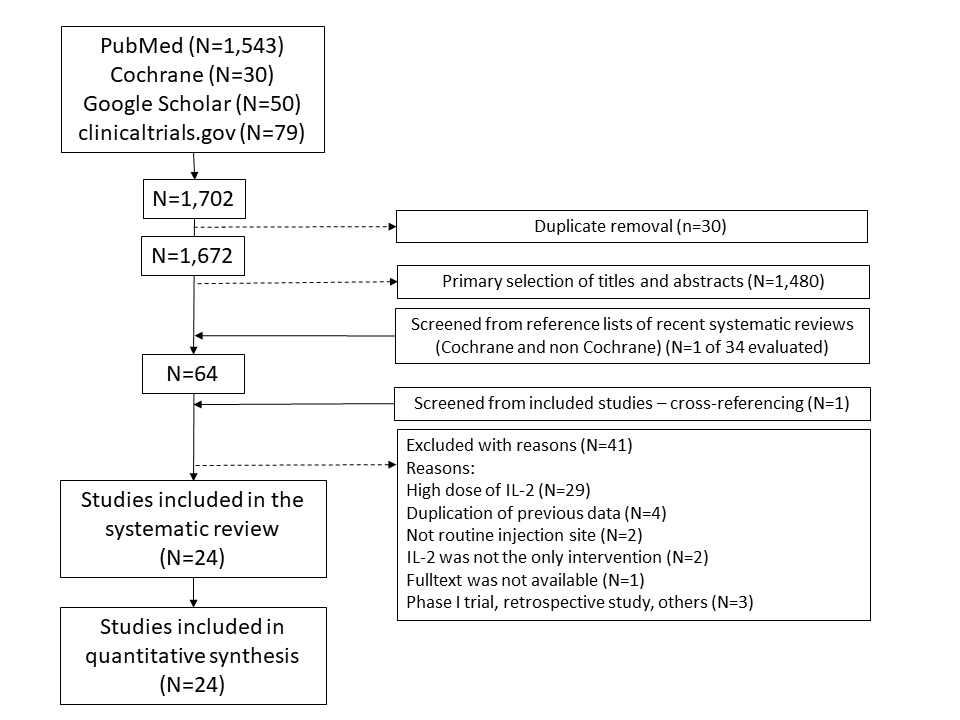
**

**Supplementary Figure S2a and S2b. Quality assessment of the included studies.**

This approach assesses risk of bias in the following six domains: sequence generation, allocation concealment, blinding (patients, clinicians, and outcome assessors), incomplete outcome data, selective outcome reporting, and other potential biases; for each area, we reported the risk of bias as one of the following: a) low risk of bias (plausible bias unlikely to seriously alter the results) if all criteria were met, b) unclear risk of bias (plausible bias that raises some doubt about the results) if one or more criteria were assessed as unclear, or c) high risk of bias (plausible bias that seriously weakens confidence in the results) if one or more criteria were not met. The assessment comprised a description and a judgment in a "Risk of bias" table, addressing specific features of the study ^6^. Differences in risk evaluation from other reviews evaluating the same articles are due to different outcomes being assessed.

**
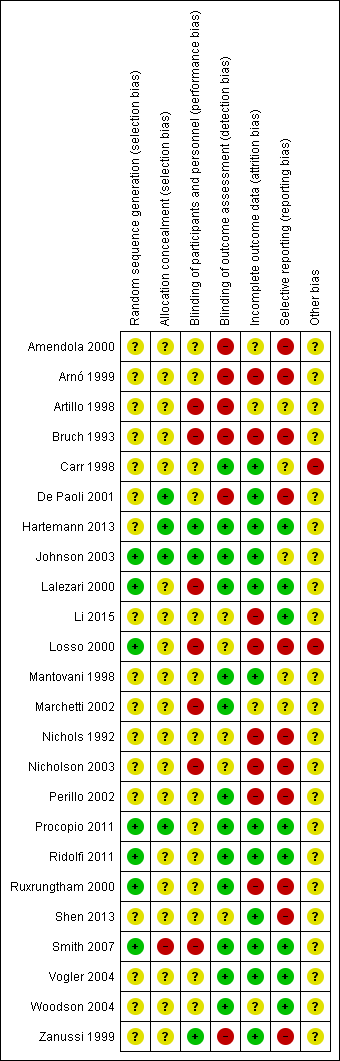
**

**
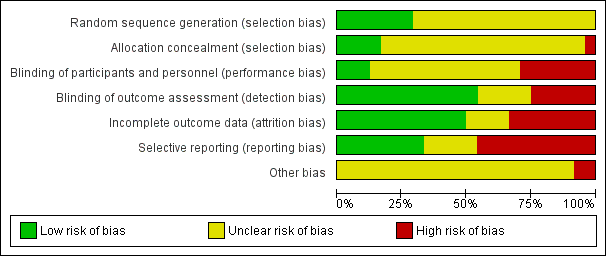
**

**Supplementary Figure S3. Risk difference of thromboembolic events.**

**
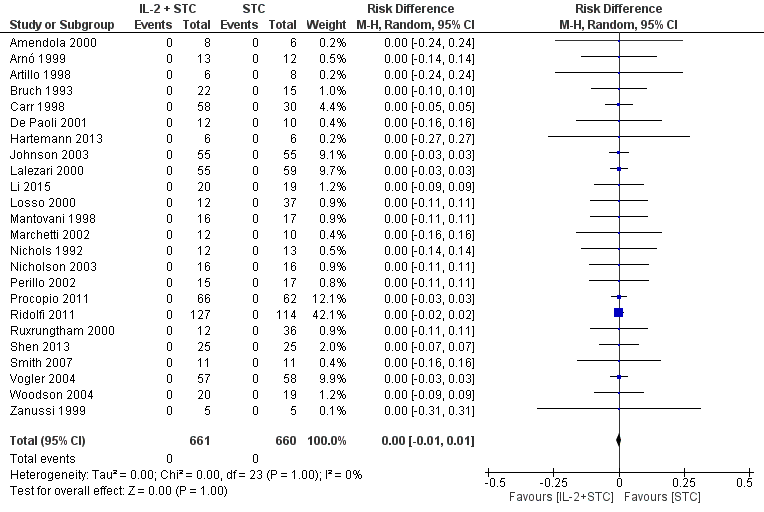
**

**Literature search strategies**

("aldesleukin"[title] OR "aldesleukin"[title] OR "proleukin"[title] OR "interleukin 2"[title]) AND ("clinical trials as topic"[MeSH Terms] OR "trial"[tiab] OR "clinical trial"[Publication Type])

N=1543 (23.03.2018) - PubMed

“Interleukin 2”

N=30 (11.04.2018) - Cochrane repository

**References**

1 Hartemann, A. *et al.* Low-dose interleukin 2 in patients with type 1 diabetes: a phase 1/2 randomised, double-blind, placebo-controlled trial. *Lancet Diabetes Endocrinol* **1**, 295-305, doi:10.1016/S2213-8587(13)70113-X (2013).

2 Johnson, J. L. *et al.* Randomized trial of adjunctive interleukin-2 in adults with pulmonary tuberculosis. *Am J Respir Crit Care Med* **168**, 185-191, doi:10.1164/rccm.200211-1359OC (2003).

3 Li, Y. *et al.* Interleukin-2 administration after modified radical mastectomy in breast cancer therapy increases peripheral regulatory T cells. *Int J Clin Exp Med* **8**, 7816-7822 (2015).

4 Shen, H. *et al.* The beneficial effects of adjunctive recombinant human interleukin-2 for multidrug resistant tuberculosis. *Arch Med Sci* **11**, 584-590, doi:10.5114/aoms.2015.52362 (2015).

5 Vogler, M. A. *et al.* Daily low-dose subcutaneous interleukin-2 added to single- or dual-nucleoside therapy in HIV infection does not protect against CD4(+) T-cell decline or improve other indices of immune function: Results of a randomized controlled clinical trial (ACTG 248). *Jaids-J Acq Imm Def* **36**, 576-587, doi:Doi 10.1097/00126334-200405010-00005 (2004).

6 Higgins, J. P. *et al.* The Cochrane Collaboration's tool for assessing risk of bias in randomised trials. *BMJ* **343**, d5928, doi:10.1136/bmj.d5928 (2011).
